# Four-Year Teriparatide Followed by Denosumab *vs.* Continuous Denosumab in Glucocorticoid-Induced Osteoporosis Patients With Prior Bisphosphonate Treatment

**DOI:** 10.3389/fendo.2021.753185

**Published:** 2021-09-27

**Authors:** Yasuaki Hirooka, Yuji Nozaki, Saki Okuda, Masafumi Sugiyama, Koji Kinoshita, Masanori Funauchi, Itaru Matsumura

**Affiliations:** ^1^ Department of Rheumatology, Kindai University Nara Hospital, Nara, Japan; ^2^ Department of Hematology and Rheumatology, Kindai University School of Medicine, Osaka, Japan

**Keywords:** bone mineral density, teriparatide, denosumab, bisphosphonate, glucocorticoid-induced osteoporosis

## Abstract

**Objectives:**

In our previous 24-month study, we observed that teriparatide had some advantages over denosumab for bone mineral density (BMD) in glucocorticoid-induced osteoporosis (GIO) patients with prior bisphosphonate treatment. We conducted this extension study to investigate whether the advantage of teriparatide obtained in the first 2 years would be maintained after the switch to denosumab.

**Materials and Methods:**

We switched patients who had completed 24-month daily teriparatide treatment to denosumab (switch group, n=18) and compared their BMD every 6 months up to 48 months with the group who continued to receive denosumab (denosumab group, n=16).

**Results:**

At 48 months, the lumbar spine BMD was significantly increased from baseline in both groups (denosumab: 10.4 ± 8.7%, p<0.001; switch: 14.2 ± 6.8%, p<0.001). However, a significant increase in femoral neck BMD from baseline occurred only in the switch group (11.2 ± 14.6%, p<0.05); denosumab (4.1 ± 10.8%). The total hip BMD increased significantly from baseline in both groups (denosumab: 4.60 ± 7.4%, p<0.05; switch: 7.2 ± 6.9%, p<0.01). Femoral neck BMD was significantly increased in the switch *versus* the denosumab group (p<0.05).

**Conclusion:**

In GIO patients with prior bisphosphonate treatment, the advantage of teriparatide may be maintained after the treatment period. A continuous increase in BMD can be expected with teriparatide followed by denosumab.

## Introduction

Glucocorticoid-induced osteoporosis (GIO) is a common and serious adverse effect associated with glucocorticoid use. GIO is characterized by decreased bone formation due to the increased apoptosis of osteoblasts and osteocytes (1, 2). A fragility fracture occurs in 30%–50% of patients who undergo long-term glucocorticoid therapy, leading to worse life expectancy and quality of life (3, 4). The most commonly used drugs for GIO are bisphosphonates, and in several randomized controlled trials, the bisphosphonates alendronate, risedronate, and zoledronate were shown to increase the lumbar and femoral bone mineral density (BMD) of GIO patients (5–7). Alendronate and risedronate were also shown to significantly reduce the rate of vertebral fractures in patients with GIO (5, 6), and zoledronic acid was shown to increase the BMD in the lumbar spine and femur to a greater degree than risedronate (7). However, even after the administration of a bisphosphonate, the BMD of some patients does not improve. Although BMD reduction alone should not be considered a failure of treatment with bisphosphonates (8), BMD is an important predictor of fractures and is one of the indicators in considering whether GIO treatment should be changed. In GIO patients whose BMD does not improve after treatment with a bisphosphonate, there is limited evidence regarding which subsequent treatment can be recommended for increasing BMD.

Denosumab, which is a RANKL (receptor activator of nuclear factor kappa-B ligand) inhibitor, and teriparatide (i.e., recombinant human parathyroid hormone (1–34)), are drugs that are expected to increase the BMD of women with postmenopausal osteoporosis more effectively than bisphosphonates (9, 10). Denosumab and teriparatide have also been shown to be effective for GIO, and they were demonstrated to increase the lumbar spine BMD and hip BMD to a greater degree compared to bisphosphonates in several studies (11–13). In the Denosumab And Teriparatide Administration (DATA) extension study of patients with postmenopausal osteoporosis — which described excellent therapeutic effects of a combination of denosumab and teriparatide — the increases in the lumbar spine, femoral neck, and total hip BMD did not differ significantly between the denosumab-monotherapy group and the teriparatide-monotherapy group after 24 months of treatment (14).

However, our study of patients with GIO showed that, unlike the DATA extension study, denosumab and teriparatide did not have equivalent effects on BMD (15). In that study, we compared the effects of teriparatide and denosumab in GIO patients who achieved low T-scores (< −2.5) in the lumbar spine or femoral neck even after bisphosphonate treatment. We observed that at 24 months after patients were switched from a bisphosphonate to denosumab or daily teriparatide, the BMD in the lumbar spine increased significantly from baseline in both groups, and there was a significant increase in the femoral neck BMD only in the teriparatide group. We thus suspected that teriparatide might have some advantages over denosumab for treating GIO patients with prior bisphosphonate treatment. However, since the clinical use of teriparatide is limited to 24 months, GIO treatment must be modified after the completion of teriparatide therapy.

The later DATA-switch study of postmenopausal osteoporosis patients revealed that the transition from teriparatide to denosumab further increased the BMD increased by teriparatide (16). The efficacy of this sequential treatment has not been well studied in GIO. In the present 4-year study, we extended our 2-year study (15) and compared a treatment group that transitioned from teriparatide to denosumab with a treatment group that continued denosumab for 4 years. We investigated whether the teriparatide advantage gained in the first 2 years would be maintained in the subsequent 2 years.

## Subjects and Methods

### Study Design

This study was conducted from 2014 to 2021 at Kindai University Hospital (Osaka, Japan). The original study (15) was a 24-month, prospective, open-label, non-randomized clinical trial. The present study’s inclusion and exclusion criteria were the same as those of the original study. GIO patients being treated with glucocorticoids for connective tissue disease and low T-score BMD (< −2.5) in the lumbar spine or femoral neck after ≥2 years of bisphosphonate therapy were switched from the bisphosphonate to either denosumab or teriparatide.

Forty-seven patients were enrolled in the original study, and 20 of 24 patients who received denosumab and 21 of 23 patients who received teriparatide completed 2 years of treatment. In the present 2-year extension study, the patients who were treated with denosumab in the original study (n=20) received an additional 24 months of denosumab (60 mg subcutaneous injection, 1×/6 months). The patients who had received daily teriparatide (n=21) were switched to denosumab. In both groups, the patients also received elemental calcium or vitamin D during the administration of denosumab.

This study was conducted according to the principles expressed in the Declaration of Helsinki of 1983, and it was approved by the Research Ethics Committee of Kindai University of Medicine. Written informed consent to participate and have their data published was obtained from all patients.

### Assessments

The demographic characteristics recorded at baseline included the patient’s age, sex, body mass index (BMI), and daily dose of prednisolone (PSL). During the extended 2-year period, as in the original study, the patients were examined every 6 months. At months 30, 36, 42, and 48 from baseline, the BMD of each patient’s lumbar spine (L1–L4) and femoral neck and total hip of the non-dominant leg were measured by dual-energy x-ray absorptiometry (Discovery A, Hologic, Marlborough, MA, USA). A marker of bone resorption, i.e., tartrate-resistant acid phosphatase 5b (TRACP5b), a marker of bone formation, i.e., procollagen type 1 N-terminal propeptide (P1NP), and albumin-corrected calcium were similarly assessed at months 30, 36, 42, and 48. The primary endpoint of this study was the percent change in BMD from the baseline of the original study to 48 months. The secondary endpoints were the percent changes in the bone turnover markers TRACP5b and P1NP every 6 months.

### Safety

The treating physicians performed the physical examinations and laboratory tests (hematological, blood chemistry, and urinalysis). All adverse events were recorded.

### Statistical Analyses

We used GraphPad Prism software (GraphPad Software, San Diego, CA) for all statistical analyses. The baseline characteristics of the denosumab and teriparatide groups were compared using the Mann-Whitney U-test (the ratio of females was tested using Fisher’s exact test). Similarly, the changes in the BMD and bone turnover markers were compared between the two patient groups by the Mann-Whitney U-test. Within-group changes in the BMD and bone turnover markers were assessed by paired t-test. P-values<0.05 were considered significant.

## Results

### Baseline Characteristics and Patient Disposition

Of the 20 patients treated with denosumab in the original study, 16 patients completed 48 months of denosumab treatment (the denosumab group). The reason for discontinuation in the other four patients were death (n=2), transfer to another hospital at the patient’s request (n=1), and missing data (n=1). The cause of death in the two cases was exacerbation of the originally existing myelodysplastic syndrome in one case and newly developed lymphoma in the other.

**Figure 1:**
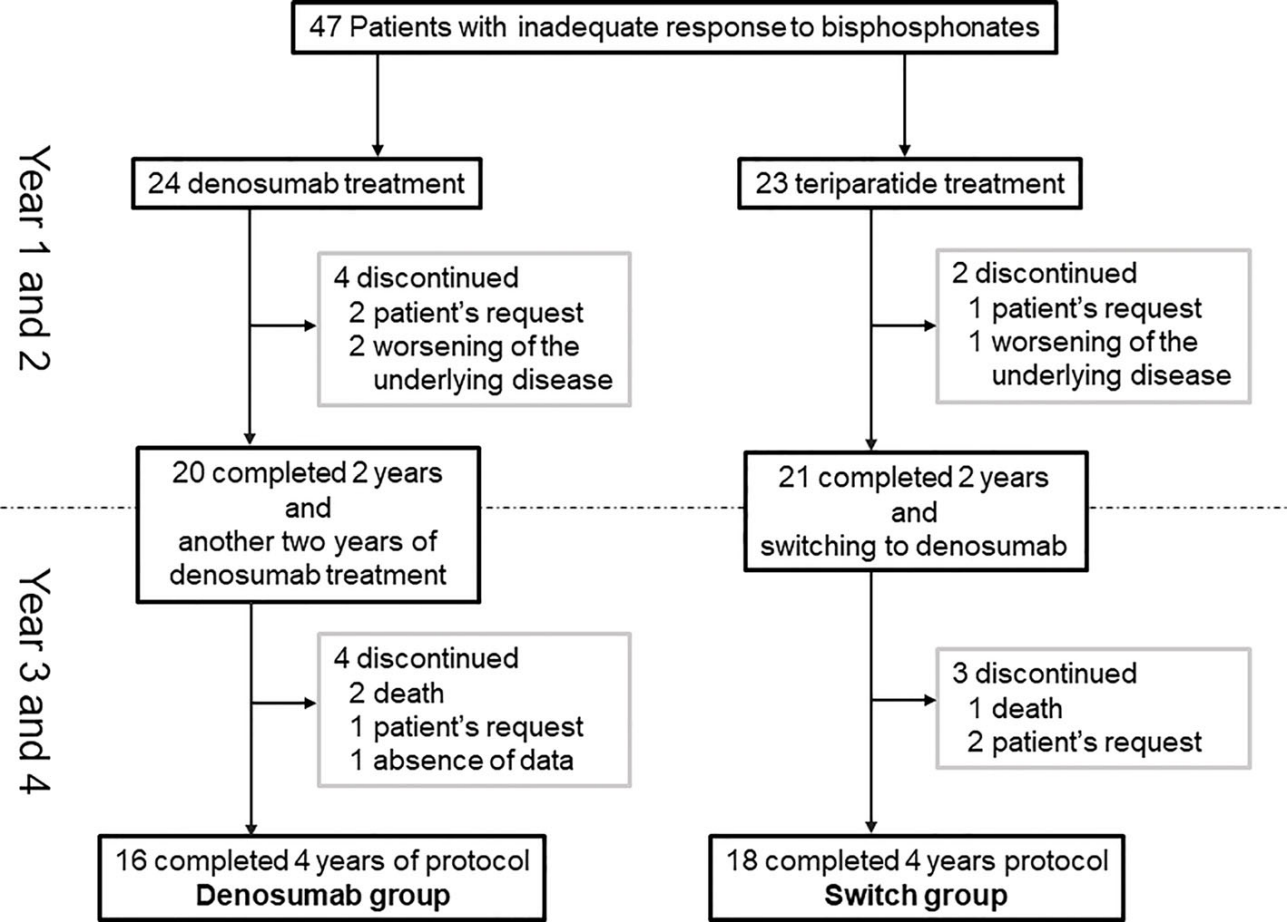
Patient enrollment and disposition.

Twenty-one patients who had been treated with teriparatide in the original 2-year study were switched to denosumab, and 18 of those patients completed a total of 48 months of treatment (the switch group). The reasons for discontinuation were hospital transfer at the patient’s request (n=1), death due to cerebral infarction (n=1), and patient request (n=1). A final total of 34 patients was analyzed (denosumab group, n=16; switch group, n=18) (**Figure 1**).

The patients’ underlying connective tissue diseases are listed in the **Supplementary Material**. The clinical characteristics of the patients at the baseline of the original study are summarized in **Table 1**. There were no significant between-group differences in age, sex, BMI, PSL dose, durations of PSL and bisphosphonate treatment, BMD, or the two bone turnover markers at baseline. No significant between-group difference was found in the daily average dose of PSL during the 48-month study period: denosumab group, 3.3 ± 2.2 mg/day; switch group, 2.9 ± 1.4 mg/day. One patient in the switch group was receiving etanercept, a tumor necrosis factor (TNF) inhibitor. No patient in either group received anti-interleukin-6 (IL-6) receptor antibody.

**Table 1:** Clinical characteristics at baseline of the original study.

Characteristics	Denosumab group	Switch group	p-value
	n = 16	n = 18	
Age, years	65.8 ± 11.3	60.3 ± 12.4	0.11
Female, %	93.8	100	0.47
BMI, kg/m^2^	20.9 ± 3.5	20.3 ± 3.0	0.56
Duration of predonisolone treatment, months	188.2 ± 106.4	201.0 ± 118.4	0.82
Dose of predonisolone at entry, mg	6.4 ± 5.1	5.0 ± 2.9	0.92
Duration of bisphosphonate treatment, months	143.2 ± 96.5	141.8 ± 79.4	0.88
BMD, g/cm2			
Lumbar spine	0.75 ± 0.12	0.74 ± 0.11	0.77
T score	-2.53 ± 1.12	-2.72 ± 1.20	0.53
Femoral neck	0.49 ± 0.08	0.50 ± 0.06	0.47
T score	-2.72 ± 0.66	-2.59 ± 0.52	0.39
Total hip	0.63 ± 0.09	0.64 ± 0.09	0.46
T score	-2.21 ± 0.70	-1.98 ± 0.86	0.46
Bone turnover markers			
Serum TRACP-5b, mU/dL	309.3 ± 116.8	253.0 ± 136.7	0.14
Serum P1NP, μg/L	32.7 ± 22.7	22.7 ± 15.7	0.13

Data are mean ± SD. BMI, body mass index; BMD, bone mineral density; TRACP-5b, tartrate-resistant acid phosphatase 5b; P1NP, procollagen type 1 N-terminal propeptide.

### Changes in BMD

**Figure 2:**
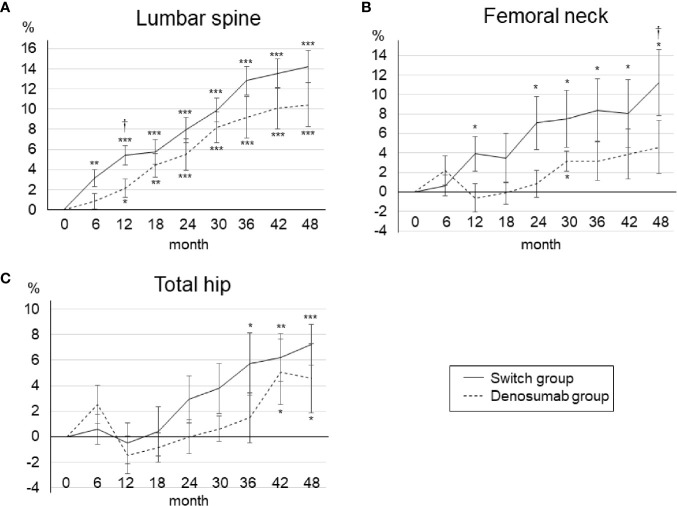
Mean percent changes in BMD from baseline to 48 months in the lumbar spine **(A)**, femoral neck **(B)**, and total hip **(C)**. Error bars: SEM. *p < 0.05, **p < 0.01, ***p < 0.001 *vs.* baseline. ^†^p < 0.05, denosumab group *vs.* switch group.

**Figure 2** illustrates the percent changes in the BMD of the lumbar spine, femoral neck, and total hip over the 48-month treatment period. Seven patients dropped out of the present study, but the results up to 24 months were roughly similar to those in the original study. The 24-month results can be summarized as follows. A significant increase occurred in the lumbar spine and femoral neck BMD from baseline in the teriparatide-treated group (which is the switch group in the present study), and a significant increase occurred in only the lumbar spine BMD in the denosumab group. At 12 months, the teriparatide-treated group showed a significant increase in the lumbar spine BMD and a tendency for a BMD increase in the femoral neck compared to the denosumab group.

At 48 months, the lumbar spine BMD had increased significantly from baseline in both groups (**Figure 2A**). The percent changes in the lumbar spine BMD from baseline to 48 months were as follows: denosumab group, 10.4 ± 8.7% (p<0.001); switch group, 14.2 ± 6.8% (p<0.001). At 48 months, there was no significant between-group difference in the lumbar spine BMD. In the femoral neck, the percent changes in BMD from baseline to 48 months were as follows: denosumab group, 4.1 ± 10.8% (p=0.21); switch group, 11.2 ± 14.6% (p<0.05) (**Figure 2B**). At 48 months, the BMD of the femoral neck was significantly increased from baseline only in the switch group, and the BMD was significantly increased in the switch group compared to the denosumab group (p<0.05). In the total hip, the percent changes in BMD from baseline to 48 months were: denosumab group, 4.60 ± 7.4% (p<0.05); switch group, 7.2 ± 6.9% (p<0.01) (**Figure 2C**). At 48 months, there was no significant between-group difference in the total hip BMD.

Compared to 24 months, the BMD in the denosumab group at 48 months was significantly increased in both the lumbar spine and total hip. In the switch group, compared to 24 months, the BMD at 48 months was significantly increased at all measurement sites. The percent changes in BMD from 24 to 48 months were not significantly different between the two treatment groups at any of the measurement sites.

As shown in the original study, a clinical vertebral fracture occurred in two patients in the denosumab group during the first 2 years. During the extended 2-year period, no new clinical fractures occurred in either patient group.

### Changes in the Bone Turnover Markers and Calcium

**Figure 3:**
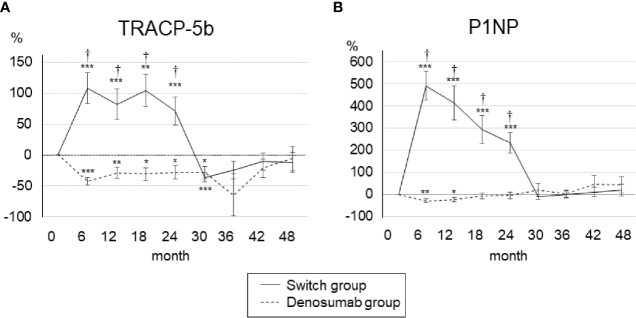
Percent changes in serum TRACP-5b **(A)** and P1NP **(B)** from baseline to 48 months. Error bars: SEM. *p < 0.05, **p < 0.01, ***p < 0.001 *vs.* baseline. ^†^p < 0.001, denosumab group *vs.* switch group. TRACP-5b, tartrate-resistant acid phosphatase 5b; P1NP, procollagen type 1 N-terminal propeptide.

The changes in bone turnover markers are shown as a percentage change from baseline in **Figure 3**. In the denosumab group, the serum TRACP-5b levels were decreased the most at 6 months (−42.1 ± 6.2%) compared to baseline and significantly decreased until 30 months, and the serum P1NP levels were decreased the most at 6 months (−30.4 ± 8.3%) compared to baseline and significantly decreased until 12 months. In the switch group, both the serum TRACP-5b and serum P1NP levels increased the most at 6 months of teriparatide treatment compared to baseline (108.4 ± 25.1% and 491.6 ± 66.5%, respectively) and increased significantly until 24 months.

After the switch from teriparatide to denosumab, the serum TRACP5-b and serum P1NP levels decreased sharply at 30 months (−110.5 ± 26.9% and −12.3 ± 12.1%, respectively), and after 30 months there was no significant difference from baseline. During the first 24 months, the serum TRACP-5b and serum P1NP levels in the switch group were significantly increased compared to those of the denosumab group, but after 30 months, there was no significant difference between the two groups. There were no clinically meaningful changes in albumin-corrected calcium in the two groups (**Supplementary Material**).

### Adverse Events

During the period of 24 to 48 months, one case each of ischemic enteritis, urinary tract infection, myocardial infarction, and ovarian tumor were reported in the denosumab group, and one case each of herpes zoster and angina pectoris were reported in the switch group. These adverse events were classified as unrelated to treatment by each patient’s physician and the study investigators.

## Discussion

In the results of our 4-year study, treatment with teriparatide for 2 years followed by denosumab for 2 years significantly increased BMD from baseline in the lumbar spine, femoral neck, and total hip in GIO patients with prior bisphosphonate treatment. Continuous treatment with denosumab for 4 years also significantly increased BMD in lumbar spine and femoral neck, but the increase in femoral neck BMD was significantly greater with teriparatide followed by denosumab. Both denosumab and teriparatide are the effective agents to increase BMD in GIO patients. However, there are few reports investigating the effects of these drugs in patients with GIO who have previously been treated with bisphosphonates, and no study has compared the two drugs in such patients. Our original study compared the efficacy of both drugs in GIO patients with prior bisphosphonate treatment, and this extension study investigated effective long-term treatment strategies for GIO with these drugs.

There are only a few studies comparing the therapeutic effects of denosumab and teriparatide. In the DATA extension study, which was conducted in bisphosphonate-naïve women with postmenopausal osteoporosis, both the denosumab- and teriparatide-treated groups showed significant increases from baseline in BMD at the lumbar spine, femoral neck, and total hip, with no significant differences between the two groups (14). On the other hand, our earlier study demonstrated that teriparatide has some advantages over denosumab in GIO patients with prior bisphosphonate treatment (15). The discrepancy in the results may be due to the different backgrounds of the patients addressed in each study. Osteoporotic patients who have been treated with bisphosphonates have already had their bone turnover sufficiently suppressed, and it is possible that the therapeutic effect of denosumab (which suppresses bone turnover like bisphosphonates do) is restricted. In addition, since GIO is caused primarily by an inhibition of bone formation, we considered teriparatide, a bone-forming agent, appropriate for the treatment of GIO.

Denosumab and teriparatide are potent osteoporosis therapeutic agents that produce large increases in lumbar and femoral BMD values. However, the discontinuation of either of these drugs results in a rapid decline in BMD (17–19). Since the clinical use of teriparatide is limited to 24 months, an important issue must be addressed: how to maintain or further increase the BMD gain that was obtained during this period. The DATA-switch study of postmenopausal women with osteoporosis reported that the transition from teriparatide to denosumab showed a greater increase in BMD than the transition from denosumab to teriparatide (16). The order of the administration of denosumab and teriparatide may affect the outcome of increased BMD, but this has not been fully examined in GIO patients. As with postmenopausal osteoporosis, teriparatide followed by denosumab would lead to a favorable increase in the BMD of patients with GIO. In our results, femoral neck BMD at 48 months was significantly increased in the switch group compared to the denosumab group. This suggest that the BMD increase achieved with teriparatide may be greater with a subsequent denosumab administration, and that the advantage of teriparatide over denosumab may be maintained after the switch to denosumab.

The body’s BMD depends on the balance between bone resorption and bone formation. In the switch group, both serum TRACP-5b, measured as a bone resorption marker, and serum P1NP, measured as a bone formation marker, were increased by teriparatide treatment, and these markers’ values then decreased after the switch to denosumab. In the present denosumab group, both serum TRACP-5b and serum P1NP were suppressed, and the patients’ serum levels of TRACP-5b decreased significantly compared to the baseline for a longer period than the serum P1NP. These changes in bone turnover markers may be related to the increase in BMD in both groups; however, changes in these markers alone may not be sufficient to explain the difference in the BMD results between the two groups. Switching from bisphosphonates to denosumab suppresses bone turnover markers in postmenopausal osteoporosis patients (20–22), but there are no long-term data over 4 years, and data in GIO patients are also insufficient. In our results, bone turnover markers in the denosumab group were reduced from baseline, but these suppressions were less than would be expected from previous reports. Although the exact cause of these discrepancies is unknown, there were differences in baseline characteristics of patients between our study and previous reports in that our study had a longer duration of treatment with bisphosphonates.

The dose of glucocorticoids used in inflammatory or autoimmune diseases depends on each disease and its severity. Strong immunosuppressive therapy for vasculitis and systemic lupus erythematosus requires high doses of glucocorticoids, whereas the use of ≤4 mg/day of PSL is often sufficient to improve symptoms in rheumatoid arthritis (23, 24). Because glucocorticoids increase the risk of BMD loss and fracture in a dose-related manner (25), differences in the underlying disease may affect the efficacy of therapeutic agents. Patients with various connective tissue diseases were included in the present study, but there was no significant difference in glucocorticoid dosage between the denosumab and switch groups.

In addition to long-term administration, the effects of glucocorticoids on bone metabolism are also observed in the short term. Even if administered for only a few days, high doses of glucocorticoids can affect bone metabolic markers and also cause increased serum intact parathyroid hormone (PTH) levels and urinary calcium excretion (26). In the present study, both the denosumab and teriparatide groups did not use more than 20 mg/day of PSL during the observation period, and there was no significant difference in the daily average dose of PSL between the groups. The usage of PSL in the two groups was thus considered to be similar.

This study was designed to evaluate patients with GIO, and patients were enrolled regardless of gender. Most of the patients who participated in this study were female, but one male was included in the denosumab group. The prevalence of osteoporosis is more common in women than in men. Women have a lower peak bone mass, smaller bone size, and tend to lose bone at a younger age than men (27). Excluding one male patient in the denosumab group did not significantly affect our results.

The major limitations of the present study are the lack of randomization and the small sample size. In the original study, the patients who chose the daily subcutaneous injection and were judged by their physician to be capable of self-injection were assigned to receive teriparatide, and the other patients were assigned to receive denosumab. Although there was no apparent difference in the baseline characteristics investigated between the two groups, it is possible that a larger number of patients in the denosumab group who were judged unable to perform self-injections by their physicians also contained patients with low physical activity. In postmenopausal women, exercise is effective for preventing lumbar spine BMD loss (28), and there is a report that the combination of teriparatide and whole-body vibration exercise resulted in a greater increase in lumbar spine BMD than teriparatide alone (29). Potential differences in physical activity and the exercise habits that might accompany it between the present denosumab and switch groups could have affected our results.

In addition, the present study’s primary endpoint was the percent changes in BMD, not the incidence of fractures, and there were no regular radiographic examinations to identify fractures. It is not sufficient to determine the treatment effect solely by measuring the BMD without assessing the incidence of fractures; however, since BMD is an important predictor of fracture, we believe that the present evaluation of the changes in BMD is very meaningful.

In the final results of our study, the 4-year treatment with teriparatide followed by denosumab in GIO patients with prior bisphosphonate treatment resulted in a continuous and large increase in BMD in the lumbar spine and femur. Since glucocorticoid therapy for connective tissue diseases is long-term, continuous therapy for GIO is also necessary. It is desirable to judge the effects of a therapeutic drug for GIO by referring to changes in BMD and bone turnover markers; in addition, patients who are considered to have an inadequate response to bisphosphonates should be considered for a switch to an appropriate agent. Our findings are important for rational drug selection in the long-term continuum of drug therapy for GIO.

## Conclusions

In our 4-year study, treatment with teriparatide followed by denosumab significantly increased lumbar and femoral BMD values, with a greater increase in femoral neck BMD than treatment with continued denosumab. The advantage of teriparatide over denosumab in GIO patients who received bisphosphonate as a pretreatment may be maintained after the teriparatide treatment period, and treatment with teriparatide first and then with denosumab is expected to result in a continued BMD gain. Further studies with larger patient populations are needed to confirm the efficacy of this treatment strategy.

## Data Availability Statement

The raw data supporting the conclusions of this article will be made available by the authors, without undue reservation.

## Ethics Statement

The studies involving human participants were reviewed and approved by The Research Ethics Committee of Kindai University of Medicine. The patients/participants provided their written informed consent to participate in this study.

## Author Contributions

YH and YN designed the study. YH and SO collected and analyzed the data. YH drafted the manuscript. YN, MS, KK, MF, and IM edited the manuscript. All authors contributed to the article and approved the submitted version.

## Conflict of Interest

YN has received honoraria or a research grant from Daiichi Sankyo and Eli Lilly Japan K.K.

The remaining authors declare that the research was conducted in the absence of any commercial or financial relationships that could be construed as a potential conflict of interest.

## Publisher’s Note

All claims expressed in this article are solely those of the authors and do not necessarily represent those of their affiliated organizations, or those of the publisher, the editors and the reviewers. Any product that may be evaluated in this article, or claim that may be made by its manufacturer, is not guaranteed or endorsed by the publisher.
