# Supplementary material for: Four-Year Teriparatide Followed by Denosumab vs. Continuous Denosumab in Glucocorticoid-Induced Osteoporosis Patients With Prior Bisphosphonate Treatment
Source: Front Endocrinol (Lausanne). 2021 Sep 27;12:753185. doi: 10.3389/fendo.2021.753185 (PMC8503555; doi:10.3389/fendo.2021.753185)
Supplement: Supplementary file 1 [file DataSheet_1.docx]

Supplementary Material

## Supplementary Table S1

ANCA: antineutrophil cytoplasmic antibody, RS3PE: remitting seronegative symmetrical synovitis with pitting edema.

## Supplementary Figure S1


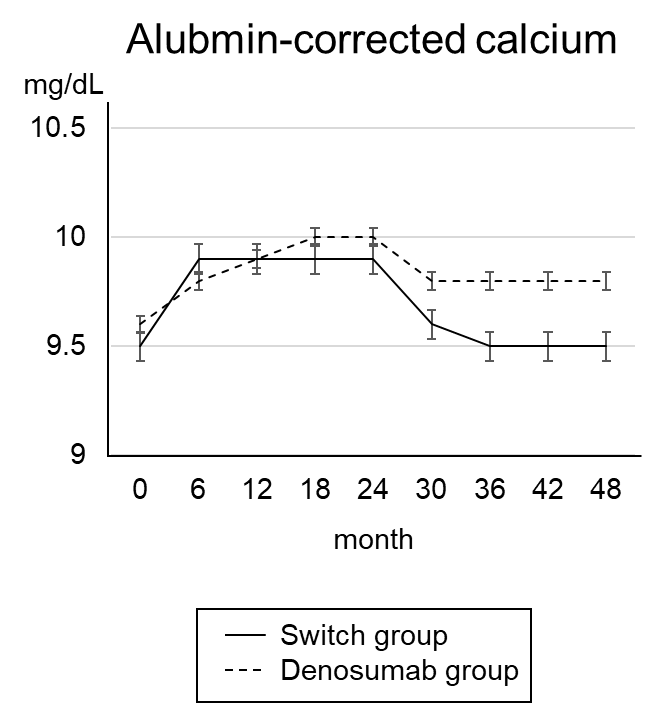


**Supplementary Figure S1.** The levels of albumin-corrected calcium from baseline to 48 months. Error bars: SEM.
